# Long non-coding RNA MEG3 functions as a competing endogenous RNA to regulate gastric cancer progression

**DOI:** 10.1186/s13046-015-0197-7

**Published:** 2015-08-08

**Authors:** Weizhao Peng, Shuang Si, Qingxia Zhang, Chaofeng Li, Fang Zhao, Fang Wang, Jia Yu, Ren Ma

**Affiliations:** Department of General Surgery, China-Japan Friendship Hospital, Beijing, 100029 China; Department of Obstetrics and gynecology, China-Japan Friendship Hospital, Beijing, 100029 China; Department of Biochemistry, Institute of Basic Medical Sciences, Chinese Academy of Medical Sciences (CAMS) & Peking Union Medical College (PUMC), Beijing, 100005 China

**Keywords:** MEG3, Gastric cancer, Competing endogenous RNA, miR-181a

## Abstract

**Background:**

Long noncoding RNAs (lncRNAs) have recently emerged as important regulators in governing fundamental biological processes, and many of which are likely to have functional roles in tumorigenesis. Maternally expressed gene 3 (MEG3) gene encodes a lncRNA whose expression is lost in an expanding list of primary human tumors and tumor cell lines, however its biological role and regulatory mechanism in gastric cancer (GC) development and progression are poorly defined.

**Methods:**

Quantitative RT-PCR analysis was used to determine whether aberrant MEG3 expression was associated with GC patients pTNM stage and pM state. Furthermore, the effect of ectopic expression of MEG3 on cell proliferation, migration, invasion and cell apoptosis was assessed by using CCK-8, wound healing, transwell invasion assays and flow cytometric analysis, respectively, in GC cell lines HGC-27 and MGC-803. Moreover, the competing endogenous RNA (ceRNA) activity of MEG3 on miR-181a was investigated via luciferase reporter assay and immunoblot analysis.

**Results:**

MEG3 is decreased in GC patients and cell lines, and its expression was associated with metastatic GC. Furthermore, ectopic expression of MEG3 in HGC-27 and MGC-803 cells inhibited cell proliferation, migration, invasion, and promoted cell apoptosis, which might be due to MEG3 sequestering oncogenic miR-181 s in GC cells. Furthermore, MEG3 could up-regulated Bcl-2 via its competing endogenous RNA (ceRNA) activity on miR-181a.

**Conclusions:**

These findings suggest that lncRNA MEG3, a ceRNA of miR-181 s, could regulate gastric carcinogenesis and may serve as a potential target for antineoplastic therapies.

## Introduction

The ENCODE (encyclopedia of DNA elements) project showed that ~80 % of the human genome is transcribed to RNA, with only ~2 % being responsible for protein coding. According to their size, non-coding RNAs (ncRNAs) are classified into small ncRNAs and long ncRNAs (lncRNAs). Small ncRNAs include siRNAs, piRNAs, and miRNAs that have a length of less than 200 nucleotides (nt). LncRNAs are greater than 200 nt in length, frequently up to hundred kb. Recent studies have revealed that a number of lncRNAs have essential roles in a diverse range of cellular functions such as development, differentiation, and cell fate as well as disease pathogenesis, causing a paradigm change in our understanding of gene regulation [1–3]. For example, the metastasis-associated lungadenocarcinoma transcript1 (MALAT1), also known as NEAT2, has been implicated in several studies as having an important role in metastasis [4–6]. H19 dysregulation has also been implicated in a variety of other cancers, as colorectal cancer, hepatocellular carcinoma, breast cancer, and bladder cancer [7–11]. Additionally, the HOX antisense intergenic RNA (HOTAIR) was shown to be overexpressed up to 2000 fold in breast cancer metastases, with its expression being a significant predictor of metastasis and death independent of other risk factors such as tumor size, stage, and hormone receptor status [12–14]. LncRNA, colon cancer associated transcript 2 (CCAT2), was found to have increased expression in metastatic colorectal cancer patient tumor samples. The lncRNA gastric cancer associated transcript 1 (GACAT1), was found to be expressed at lower levels in gastric cancer tissues compared to corresponding normal tissues [15, 16]. Identification of differential expression, while extremely useful, is only the first step in the elucidation of the lncRNA-based molecular mechanisms capable of regulating tumorigenesis. Exploration of the function and involvement of lncRNA in gene expression may become a key development in exploring the molecular mechanisms of cancer.

Gastric cancer (GC) ranks the fourth most commonly diagnosed cancer and the second lethal malignancy worldwide, and is the most common gastrointestinal malignancy in East Asia, Eastern Europe, and parts of Central and South America [17–19]. GC is diagnosed at advanced stage accompanied by malignant proliferation and metastasis. In spite of the progress in chemotherapy, radiotherapy and surgical techniques for GC in recent years, the survival rate of GC patients remains unsatisfactory [20]. Although many oncogenes or tumor suppressors have been identified as key players underlying tumorigenesis of GC, however, almost no commonly-accepted biomarkers have been established to facilitate the comprehensive management of patients [21, 22]. Therefore, the identification of the new regulators and therapeutic targets for GC and a detailed understanding of the molecular mechanisms underlying gastric carcinogenesis will be important to understand the molecular biology of tumor and its progression. The significance of lncRNAs in human GC was realized by Yang and colleagues elucidating the contributions of H19 to GC, suggesting a link between lncRNAs and GC [7]. Following this study, several groups focused on the aberrant expression of lncRNAs during GC, and accumulating studies indicated that specific lncRNAs had potential biological and clinical relevance in GC [23]. Therefore, it is of great clinical value to identify cancer-associated lncRNAs and investigate their molecular and biological functions for GC prevention, diagnosis, and therapeutic targets.

In the current study, we showed that a lncRNA, maternally expressed gene 3 (MEG3) is decreased in GC patients and cell lines, and its expression was associated with metastatic GC. We also showed that MEG3 inhibited GC cell proliferation, migration and invasion by operating as a competing endogenous RNA (ceRNA) for the miR-181 microRNA (miRNA) family. Furthermore, MEG3 affected GC cell phenotypes in a miR-181 sites-dependent manner, which occurs without changes in the levels of miR-181 isoforms, suggesting that MEG3 regulates miR-181 activity by altering miRNA targeting. B cell lymphoma-2 (Bcl-2) was subsequently validated as a downstream target of MEG3 ceRNA function, and was important for MEG3 to regulate GC progression. Taken together, these results suggest that MEG3 could regulate gastric carcinogenesis as a ceRNA and may serve as a potential target for antineoplastic therapies.

## Materials and methods

### Gastric cancer tissues

Gastric cancers and their morphologically normal tissue (located >3 cm away from the tumor)were obtained between November 2011 and November 2014 from 50 gastric cancer patients undergoing surgery at Cancer Hospital of Chinese Academy of Medical Sciences. Tissue samples were cut into two parts, one was fixed with 10 % formalin for histopathological diagnosis, and the other was immediately snap-frozen in liquid nitrogen, and stored in liquid nitrogen until RNA extraction. This group consisted of 38 males and 12 females with a median age of 58 years (range, 32–69 years). The use of the tissue samples for all experiments was approved by all the patients and by Ethics Committee of the institution.

### Tissue RNA isolation and qRT-PCR

Total RNA was extracted from the cells and tissues using Trizol reagent (Invitrogen, CA, USA), according to the manufacturer’s instructions. RT-qPCR assay was conducted to detect the level of RNA transcripts. Briefly, cDNA was synthesised by M-MLV reverse transcriptase (Invitrogen) from 5 ug of total RNA. Oligo (dT18) RT primer was used for the reverse transcription of mRNA and lncRNA. Stem-poop RT primer was used for the reverse transcription of miR-181a. Quantitative RT-qPCR was performed on the Bio-rad CFX96 real-time PCR System (Bio-rad, Foster City, CA, USA) using KAPA PROBE FAST qPCR Kits (Kapa Biosystems, MA, USA) and TaqMan probes (Invitrogen) with the following cycling conditions: 95 °C for 10 min (initial denature); then 40 cycles of 95 °C for 15 sec, 60 °C for 60 sec. The miR-33b specific forward primer sequence was designed on the basis of miRNA sequences obtained from the miRBase database. Human GAPDH and U6 snRNA were used for mRNA/lncRNA and miRNA normalization, respectively.

### Cell cultures and cell transfection

A total of 6 human gastric cancer cell lines MGC-803, HGC-27, MKN-45, SGC-7901, BGC-823 and AGS were examined in this study. The HGC-27, MKN-45, SGC-7901 cell lines were provided by American Type Culture Collection (ATCC; Manassas, VA, USA), and were maintained in RPMI 1640 medium (PAA) supplemented with 10 % FBS (PAA). The MGC-803, BGC-823 and AGS cell lines were purchased from the Cell Resource Center of Institute of Basic Medical Sciences, Chinese Academy of Medical Sciences and Peking Union Medical College (Beijing, China), and was propagated in Dulbecco’s modified Eagle medium (Gibco; Invitrogen; Life Technologies, Germany), supplemented with 10 % fetal bovine serum (FBS; PAA, Pasching, Austria) and streptomycin (100 μg/ml), penicillin (100 U/ml). The human gastric cancer cell lines HGC-27 and MGC-803 were transfected with miRNA mimic, mimic control, miRNA inhibitor, inhibitor control, (Scramble; GenePharma; Shanghai, China) at a final concentration of 25 nmol/L using DharmaFECT 1 (Dharmacon; USA) in accordance with the manufacturer’s instructions. The same cells were transfected with different MEG3 constructs at a final concentration of 2 μg/uL using Lipo2000 (Invitrogen; USA) in accordance with the manufacturer’s instructions.

### Cell proliferation assay

Hgc-27 and MGC-803 cells were incubated in 10 % CCK-8 (DOJINDO, Japan) diluted in normal culture medium at 37 °C until visual color conversion occurred. Proliferation rates were determined at 0, 24, 48, 72, 96 hours after transfection. The absorbance of each well was measured with a microplate reader set at 450nM and 630nM. All experiments were performed in quadruplicate.

### Cell apoptosis assay

In order to detect the apoptosis of HGC-27 and MGC-803 cells, flow cytometric analysis was applied with Annexin V-FITC/PI Apoptosis Detection Kit (KeyGEN Biotech, Nanjing, China) according to the manufacturer’s instructions. The acquisition and analysis were performed using MoFlow (Beckman Coulter, Atlanta, GA, USA).

### Cell migration and invasion assays

HGC-27 and MGC-803 cells were grown to confluence on 12-well plastic dishes and treated with miRNA mimics or Scramble. Then 24 hours after transfection, linear scratch wounds (in triplicate) were created on the confluent cell monolayers using a 200 μL pipette tip. To remove cells from the cell cycle prior to wounding, cells were maintained in serum-free medium. To visualize migrated cells and wound healing, images were taken at 0, 24, 48 hours. A total of ten areas were selected randomly from each well and the cells in three wells of each group were quantified.

For the invasion assays, after 24 hours transfection, 1 × 10^5^ HGC-27or MGC-803 cells in serum-free media were seeded onto the transwell migration chambers (8 μm pore size; Millipore, Switzerland) which coated with the upper chamber of an insert coated with Matrigel (Sigma-Aldrich, USA). Media containing 20 % FBS were added to the lower chamber. After 24 hours, the noninvading cells were removed with cotton wool, Invasive cells located on the lower surface of the chamber were stained with May-Grunwald-Giemsa stain (Sigma-Aldrich, USA) and counted using a microscope (Olympus, Tokyo, Japan). Experiments were independently repeated three times.

### Luciferase reporter assay

HGC-27 cells were co-transfected with 0.4 μg of pMIR constructs containing the wild type MEG3 or diverse mutant MEG3, along with 0.02 μg of the pRL-TK control vector and miR-181a mimic or mimic control. Cells were harvested 48 h post-transfection and assayed with Dual Luciferase Assay (Promega, WI, USA) according to the manufacturer’s instructions. All transfection assays were carried out in triplicate.

### Immunoblotting

Immunoblot analysis was carried out using standard methods. Proteins were separated by 10 % SDS-PAGE, and transferred onto PVDF membranes (Millipore Corporation, Billerica MA, USA). Membranes were blocked overnight with 5 % non-fat dried milk for 2 h and incubated with anti-Bcl-2 antibody (Abcam, ab117115) at 1:2000 dilution; anti-GAPDH antibody (Proteintech) at 1:50,000 dilution overnight at 4 °C. After washing with TBST (10 mM Tris, pH 8.0, 150 mM NaCl, and 0.1 % Tween20), the membranes were incubated for 2 h at room temperature with goat anti-rabbit antibody (Zsgb-bio, Beijing, China) at 1:20000.

### RNA pull-down by MS2-MBP

Maltose-binding protein (MBP)-affinity purification was used to identify miRNAs that associated with lncRNA MEG3. The MS2-MBP protein was expressed and purified from E. coli following a protocol from the Steitz lab. Three bacteriophage MS2 coat protein-binding sites (5′-cgtacaccatcagggtacgagctagcccatggcgtacaccatcagggtacgactagtagatctcgtacaccatcagggtacg-3’) were inserted downstream of MEG3 by site-directed mutagenesis using Stratagene’s QuikChange Site-Directed Mutagenesis Kit. To obtain miRNAs associated with MEG3, HGC-27 cells were transfected with MS2-containing MEG3 constructs, and 10 million cells were used for each immunoprecipitation assay. The cells were harvested 48 h post-transfection and subjected to RNA pull-down analysis as described elsewhere.

## Results

### Aberrant expression of lncRNA MEG3 in human GC tissues and cell lines

**Fig. 1:**
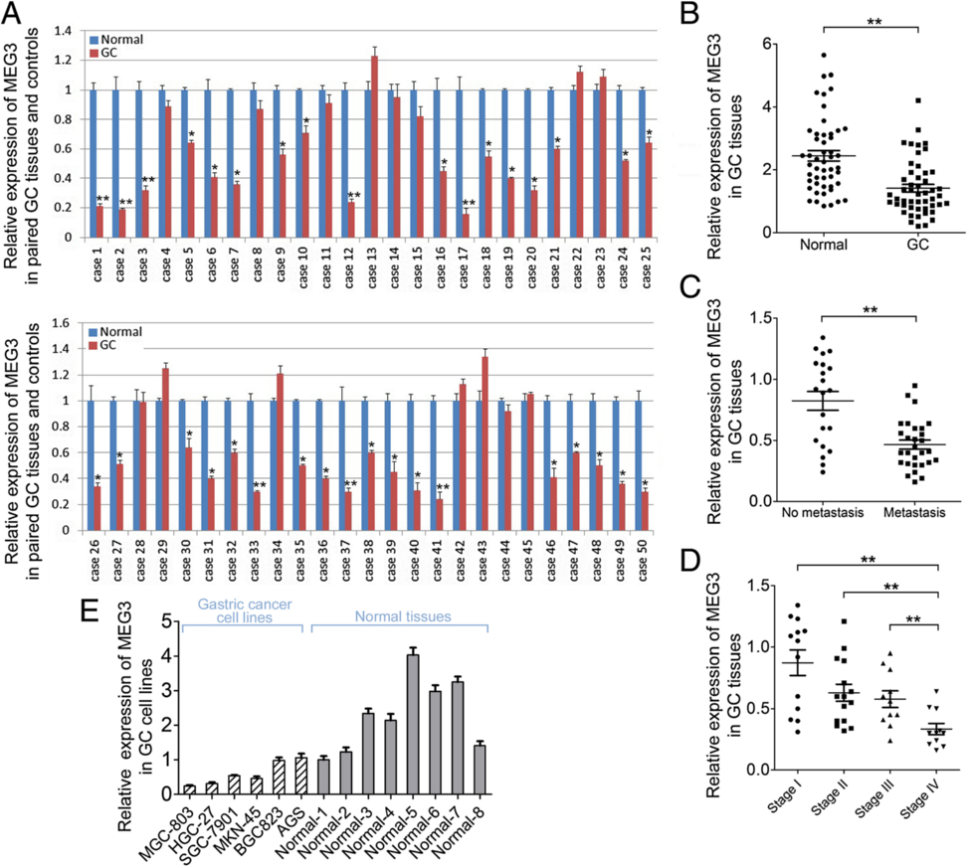
The expression of MEG3 in GC tissues and cell lines. **a** The expression of level of MEG3 was detected in 50 GC patients by RT-qPCR. Data was presented as fold change of GC tissues relative to adjacent normal regions; **b** Relative MEG3 expression level in GC tissues and adjacent normal regions; **c** The Statistical analysis of the association between MEG3 level and pM stage (No metastasis and Metastasis); **d** The Statistical analysis of the association between MEG3 level and pTNM stage (I, II, III and IV); **e** The relative level of MEG3 in GC cell lines (HGC-27,MGC-803, MKN-45, SGC-7901, BGC-823 and AGS) relative to 8 normal control samples; For all quantitative results, the data are presented as the mean ± SEM, and the error bars represent the standard deviation obtained from three independent experiments. *p < 0.05; **p < 0.01

To determine the expression of lncRNA MEG3 in GC, q-PCR analysis using TaqMan probes was conducted in 50 pairs of clinic GC tissue and matched adjacent normal tissue samples. Among them, 35 cases (70 %) showed significantly reduced level of MEG3 in tumor tissues compared with their normal tissues, whereas only 8 cases (16 %) showed up-regulated MEG3 level in GC (Fig. 1a). In summary, MEG3 was down-regulated in GC tissue compared with adjacent normal tissues (Fig. 1b). To further study the relationship between MEG3 level and clinicopathological factors of GC, the expression of MEG3 in GC tissues were statistically analyzed (non-parametric test). Interestingly, we found that the lower MEG3 was associated with pM stage (Fig. 1c, p < 0.01, metastasis *vs.* non metastasis) and pTNM stage (Fig. 1d, p < 0.01, stageI*vs.* IV; p < 0.01, stage II *vs.* IV; p < 0.01, stage III *vs.* IV) in GC patients. However, there was no significant difference between the expression level of MEG3 and other clinicopathologic characteristics, including gender, age, venous invasion, position, borrmann typing, pT stage, pN stage in GC (data not shown). These results indicated that the dysregulation of MEG3 in GC patients might suggest a potential tumor suppressor role of MEG3 in GC tumorigenesis.

### MEG3 inhibits GC cell proliferation, migration and invasion

**Fig. 2:**
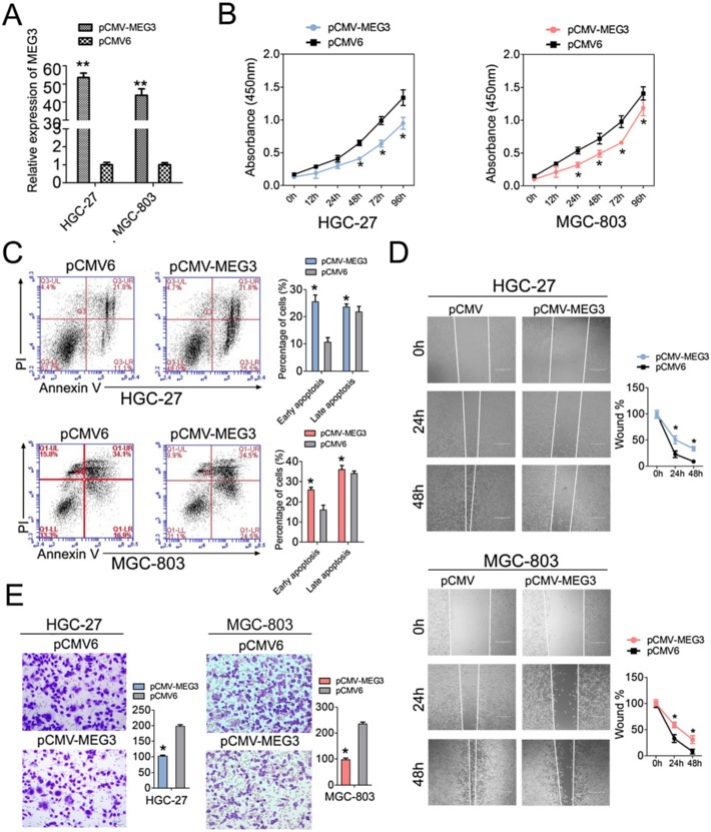
The functional analysis of MEG3 in GC cells. **a** YAP1 level were detected in HGC-27 and MGC-803 cells after treatment with pCMV-MEG3 or pCMV6 empty vector by RT-qPCR; **b** Cell proliferation assay of HGC-27 and MGC-803 cells after treatment with si-YAP1 or control by using CCK-8; **c** Flow cytometry was applied to examine the apoptosis of HGC-27 and MGC-803 cells after treatment with pCMV-MEG3 or pCMV6 and stained with apoptosis markers (FITC-Annexin V and PI). In the apoptosis map, FITC-Annexin V^+^/PI^+^ indicates late apoptosis, FITC-Annexin V^+^/PI^−^ indicates early apoptosis, and FITC-Annexin V^−^/PI^−^ indicates normal live cells. **d** Wound healing assays of HGC-27 and MGC-803 cells after treatment with pCMV-MEG3 or pCMV6, The relative ratio of wound closure per field was shown in the right; **e** Transwell analysis of HGC-27 and MGC-803 cells after treatment with pCMV-MEG3 or pCMV6, The relative ratio of invasive cells per field was shown right. For all quantitative results, the data are presented as the mean ± SEM, and the error bars represent the standard deviation obtained from three independent experiments. *p < 0.05; **p < 0.01

To test whether MEG3 play an important role in gastric carcinogenesis, the level of MEG3 in GC cell lines was first measured and the results showed that MEG3 was significantly down-regulated in MGC-803, HGC-27, SGC-7901 and MKN-45 (Fig. 1e). Of them, MGC-803 and HGC-27 were selected to study the function of MEG3. A construct containing MEG3 transcripts (pCMV-MEG3) was transfected into the HGC-27 and MGC-803 cells and the efficiency of MEG3 overexpression was subsequently confirmed by q-PCR analysis (Fig. 2a). The intracellular level of MEG3 was enhanced by 50-fold and 40-fold in HGC-27 and MGC-803 cells treated with pCMV-MEG3 than the empty vector pCMV6, respectively (Fig. 2a). Accordingly, The proliferation of transfected MGC-803 and HGC-27 cells was measured by using CCK-8 assay. Ectopic expression of MEG3 led to significant decrease in cell proliferation in both HGC-27 and MGC-803 cells (Fig. 2b). Furthermore, we examined the effects of MEG3 on the apoptosis of HGC-27 and MGC-803 cells receiving MEG3 or not with flow cytometry. The flow cytometry results showed that MEG3 increased the early and late apoptosis of HGC-27 and MGC-803 cells compared to control group (Fig. 2c).

Based on the correlation between MEG3 expression and metastatic factors, we proposed that this lncRNA might play an important role in regulating cell migration and invasion of GC cells. To test this hypothesis, cell migration and invasion assays were performed in HGC-27 and MGC-803 cells transfected with pCMV-MEG3 or pCMV6. As a result, the wound healing assay showed that cell migration was inhibited in MEG3-overexpressed GC cells compare to the controls (Fig. 2d). Moreover, transwell invasion assay indicated a significant reduction in cell invasiveness after pCMV-MEG3 transfection into both HGC-27 and MGC-803 cells (Fig. 2e). Taken together, these results suggest that MEG3 may act as a tumor suppressor through inhibiting cell proliferation, migration and invasion, and promoting cell apoptosis in GC.

### MEG3 is physically associated with miR-181 family

**Fig. 3:**
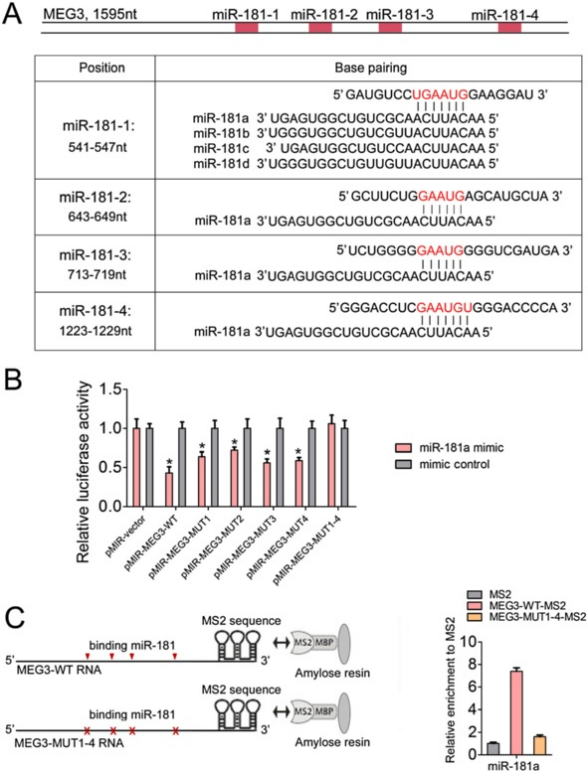
The interaction of MEG3 with miR-181. **a** The upper panel: schematic outlining the predicted binding sites of miR-181 s on MEG3. The lower panel: the prediction for miR-181 s binding sites on MEG3 transcript. The red nucleotides are the complementary sequences to miR-181 seed sequences. **b** Luciferase activity in HGC-27 cells cotransfected with miR-181a mimic (25 nM) mimic control (25 nM) and luciferase reporters containing nothing, wild type MEG3 or diverse mutant MEG3 as indicated. Data are presented as the relative ratio of firefly luciferase activity to renilla luciferase activity. **c** MS2-RIP followed by miRNA RT-qPCR to detect miR-181a endogenously associated with MEG3. For all quantitative results, the data are presented as the mean ± SEM, and the error bars represent the standard deviation obtained from three independent experiments. *p < 0.05; **p < 0.01

The ceRNA hypothesis posits that specific lncRNA can function as sinks for pools of active miRNAs, functionally liberating mRNA transcripts targeted by that set of miRNAs. To determine whether MEG3 can operate as a ceRNA, we used the webserver lnCeDB (http://gyanxet-beta.com/lncedb/) to predict potential lncRNA-miRNA interactions. Among the results, we found 4 miR-181 family binding sites scattering the MEG3 transcripts, suggesting its ceRNA potential for miR-181 s (Fig. 3a). Subsequently, we constructed a series of luciferase reporters containing the wild type MEG3 (pMIR-MEG3-WT), or mutant MEG3 with mutations of single (pMIR-MEG3-MUT1, 2, 3, 4) or all four predicted miR-181 binding sites (pMIR-MEG3-MUT1-4). The miR-181 family contains four miRNAs (miR-181a/b/c/d), which are transcribed from three separated gene loci. Here, we mainly focused on miR-181a to further investigate the interaction between MEG3 and miR-181a in GC cells. We found that transfection of HGC-27 cells with miR-181a mimic reduced the luciferase activities of the MEG3-WT reporter vector but not empty vector or all miR-181 site mutant reporter vector (Fig. 3b), suggesting the binding of miR-181a to these sites. To further validate the direct interaction between miR-181a and MEG3 at endogenous levels, we performed an RNA immunoprecipitation (RIP) analysis to pull down endogenous miRNAs associated with MEG3. The precipitated miRNAs were q-PCR analyzed and results showed that the MEG3 RIP (MEG3-WT-MS2) in HGC-27 cells was significantly enriched for miR-181a compared to the empty vector (MS2) and MEG3 with mutations in all four miR-181a targeting sites (MEG3-MUT1-4-MS2), supporting that miR-181a was bona fide MEG3-targeting miRNAs (Fig. 3c). These data demonstrated that miR-181a could bind to MEG3 and MEG3 could function as a ceRNA in GC cells.

### miR-181a function as an oncogene in GC

**Fig. 4:**
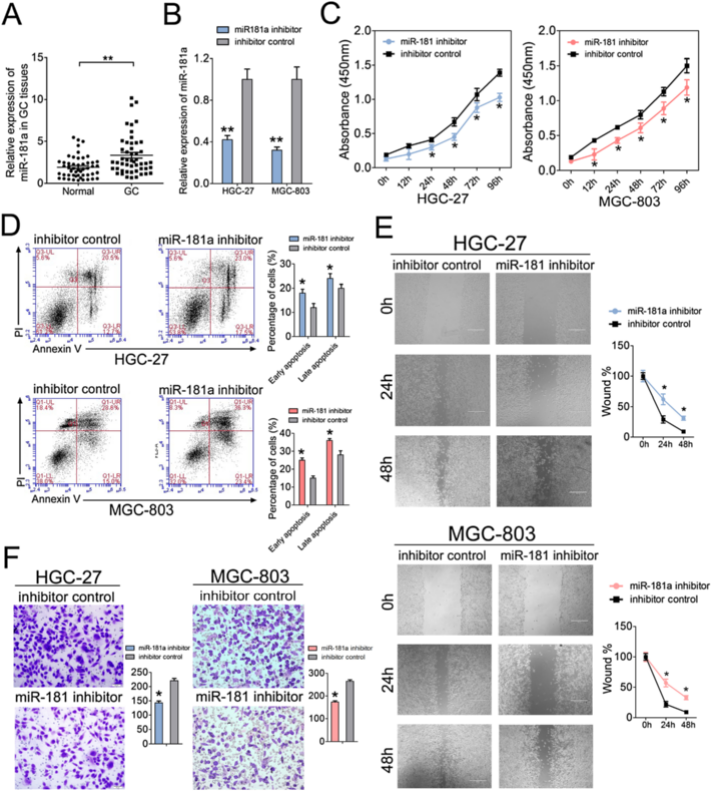
The functional analysis of miR-181a in GC cells. **a** Relative miR-181a expression level in GC tissues and adjacent normal regions; The expression of level of miR-181a was detected in 50 GC patients by RT-qPCR. **b** miR-181a level were detected in HGC-27 and MGC-803 cells after treatment with miR-181a inhibitor (25 nM) or inhibitor control (25 nM) by RT-qPCR; **c** Cell proliferation assay of HGC-27 and MGC-803 cells after treatment with miR-181a inhibitor or inhibitor control by using CCK-8; **d** Flow cytometry was applied to examine the apoptosis of HGC-27 and MGC-803 cells after treatment with miR-181a inhibitor or inhibitor control and stianed with apoptosis markers (FITC-Annexin V and PI). In the apoptosis map, FITC-Annexin V^+^/PI^+^ indicates late apoptosis, FITC-Annexin V^+^/PI^−^ indicates early apoptosis, and FITC-Annexin V^−^/PI^−^ indicates normal live cells. **e** Wound healing assays of HGC-27 and MGC-803 cells after treatment with miR-181a inhibitor or inhibitor control, The relative ratio of wound closure per field was shown in the right; **f** Transwell analysis of HGC-27 and MGC-803 cells after treatment with miR-181a inhibitor or inhibitor control, The relative ratio of invasive cells per field was shown right. For all quantitative results, the data are presented as the mean ± SEM, and the error bars represent the standard deviation obtained from three independent experiments. *p < 0.05; **p < 0.01

Based on above findings, miR-181a may also play a role in gastric carcinogenesis. We first investigated miR-181a level in above 50 pairs of clinic GC tissue and matched adjacent normal tissue samples. In contrast to MEG3, miR-181a was dramatically increased in GC tissue compared with adjacent normal tissues (Fig. 4a), indicating its oncogenic role in GC. We then used HGC-27 and MGC-803 cells to analyze the role of miR-181a. RT-qPCR was first used to measure the level of miR-181a after transfection and showed that the level of miR-181a was reduced to ~40 % and ~30 % in miR-181a inhibitor-transfected HGC-27 and MGC-803 cells (Fig. 4b). Accordingly, the CCK-8 proliferation assay in the same cells indicated that cell growth was suppressed after transfection with miR-181a inhibitor (Fig. 4c). Reduced miR-181a expression also decreased the early and late apoptosis of HGC-27 and MGC-803 cells compared to control group (Fig. 4d).

Additionally, the wound healing assay showed that cell migration was inhibited in miR-181a inhibitor-transfected HGC27 and MGC-803 cells compare to the inhibitor control-transfected ones (Fig. 4e), suggesting the stimulating effects of miR-181a on tumor cell migration. To detect whether miR-181a possesses the ability to inhibit cell invasion, transwell invasion assay was performed. As expected, there was significant reduction in cell invasiveness after miR-181a inhibitor transfection in both HGC-27 and MGC-803cell lines (Fig. 4 f). Taken together, these results indicated the oncogenic roles of miR-181a in GC cells, which was opposite to its ceRNA MEG3.

### MEG3 ceRNA activity regulates Bcl-2 expression

**Fig. 5:**
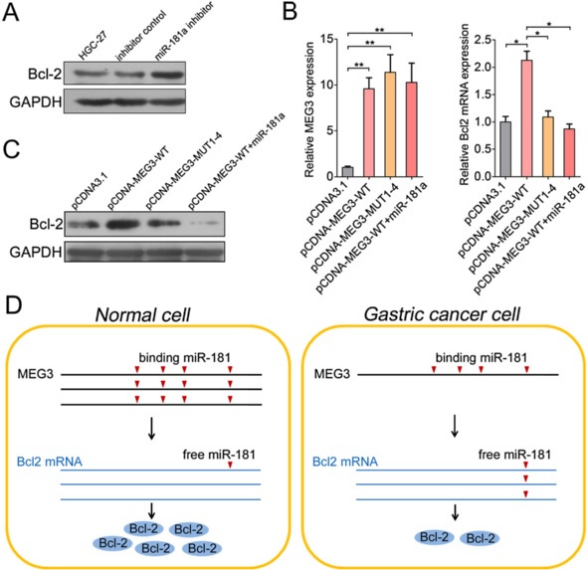
Regulation of Bcl-2 by MEG3. **a** Immunoblot analysis of Bcl-2 in HGC-27 cells transfected with miR-181a inhibitor or inhibitor control; An unrelated protein GAPDH was used as the control. **b** The expression of MEG3 and Bcl-2 mRNA in HGC-27 cells transfected with empty vector, wild type MEG3 or mutant MEG3 as indicated; **c** Immunoblot analysis of Bcl-2 protein in HGC-27 cells transfected with empty vector, wild type MEG3 or mutant MEG3 as described in B; **d** A Schematic model of MEG3/miR-181/Bcl-2 cascade in gastric carcinogenesis. For all quantitative results, the data are presented as the mean ± SEM, and the error bars represent the standard deviation obtained from three independent experiments.*p < 0.05; **p < 0.01

The oncogenic role of miR-181a promoted us to study its mechanism in gastric carcinogenesis. Studies have reported that miR-181 targets multiple Bcl-2 family members in astrocytes [24]. To validate whether miR-181a targets Bcl-2 in GC, immunoblotting assay was carried out and showed that Bcl-2 was about 2-fold higher in HGC-27 cells transfected with miR-181a inhibitor compared with the control (Fig. 5a). Because MEG3 shared regulatory miR-181a with Bcl-2 mRNA, we wondered whether MEG3 could modulate Bcl-2 in GC cells. As excepted, overexpression of wild type MEG3 (pCDNA-MEG3-WT), but not the mutant (pCDNA-MEG3-MUT1-4), increased Bcl-2 transcript (Fig. 5b) and protein levels in HGC-27 cells (Fig. 5c). Furthermore, ectopic expression of miR-181a upon pCDNA-MEG3-WT transfection abrogated this increase (Fig. 5b, c). All these results suggest an important role of MEG3 in modulating Bcl-2 by competitively binding miR-181a.

## Discussion

Maternally expressed gene 3 (MEG3) is an imprinted gene belonging to the imprinted DLK1–MEG3 locus located at chromosome 14q32.3 in human genome. Its mouse ortholog, Meg3, also known as gene trap locus 2 (Gtl2), is located at distal chromosome 12 [25]. Mice carrying the maternal deletion of the Meg3 region died perinatally and had major skeletal muscle defects. MEG3 is expressed in many normal tissues, and the loss of MEG3 expression has been found in various types of human tumors, including in 25 % of neuroblastomas, 81 % of hepatocellular cancers, and 82 % of gliomas [26, 27]. However, the involvement of MEG3 in gastric cancer has not been reported. Our findings indicated that MEG3 was down-regulated in GC tissues, and a lower level of MEG3 was associated with tumor stage and metastasis. Functional analysis confirmed the pleiotropic effects of MEG3 on GC cell proliferation, migration and invasion. Therefore, lncRNA MEG3 was determined as a novel tumor suppressor in human GC.

Although a vast set of lncRNA transcripts are differentially expressed during development where many of them play critical roles, most of them have not yet been studied in mechanistic details. Till now, a majority of the lncRNAs have been linked with epigenetic modulation of gene expressions, they can also regulate gene expression by transcriptional or post transcriptional modes. In recent years it has been discovered that endogenous lncRNAs can influence post-transcriptional regulation by interfering with the miRNA pathways, by acting as competing endogenous RNAs (ceRNAs) [28–30]. These lncRNAs have miRNA responsive elements (MRE) and act as miRNA sponges to control endogenous miRNAs available for binding with their target mRNAs, thus reducing the repression of these mRNAs [29]. It also suggests that these ceRNAs are implicated in many biological processes and the disruption of the equilibrium of ceRNAs and miRNAs can be critical for ceRNA activity and promotion of diseases like cancer. For example, lncRNA PTEN-P1 could block miR-19b and miR-20a from binding to PTEN tumor suppressor in prostate cancer, glioblastoma, and melanoma, and disruption in the network leads to tumorigenesis in many cases [28, 31]. HULC lncRNA also acts as ceRNA of the protein coding gene PRKACB that induces activation of CREB to modulate self-regulation in hepatocellular carcinoma [32]. In a recent study, lncRNA-activated by TGF-b (lncRNA-ATB) was upregulated in hepatocellular carcinoma metastases and associated with poor prognosis [33]. lncRNA-ATB upregulated ZEB1 and ZEB2 by competitively binding the miR-200 family and then induced EMT and invasion [33]. Additionally, Hmga2 promotes lung carcinogenesis both as a protein-coding gene and as a ceRNA dependent upon the presence of let-7 sites [34]. Thus, The intricate networks of ceRNAs in cells are a fascinating new subject of study for researchers working towards understanding the language of RNA molecules and gene expression networks in tumorigenesis.

In this study, we report that lncRNA MEG3 inhibits GC cell proliferation, migration and invasion by competitively binding the miR-181 family, upregulating Bcl-2, and then suppressing gastric carcinogenesis (Fig. 5d). The dysregulation of MEG3 has been reported in many cancer types and its tumor suppressor activity was mediated by interaction with either p53-dependent transcription, or Rb-related pathways. Our studies revealed a novel ceRNA activity of MEG3 in human GC cells. Although much of MEG3 ceRNA activity is driven by overexpression of Bcl-2 in GC, there are likely to be additional MEG3 ceRNA targets to be found in future studies.

## Conclusions

Taken together, our research demonstrated that MEG3 acted as a key regulator in human gastric carcinogenesis and revealed roles of MEG3 in regulating miR-181-Bcl2 axis. The findings of this study have significant implications regarding our understanding of GC pathogenesis. Moreover, the pleiotropic effects of MEG3 on GC tumorigenesis suggest that it could be an effective target for antineoplastic therapies.
